# Book Reviews

**DOI:** 10.1556/JBA.2.2013.3.9

**Published:** 2013-08-20

**Authors:** Anikó Maráz

**Affiliations:** Doctoral School of Psychology Department of Clinical Psychology and Addiction Eötvös Loránd University Budapest, Hungary


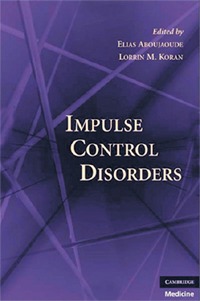


The “problem” with impulse control disorders (ICDs) is that unlike alcohol or other substance abuse, there is no saturation point. One cannot overdose simply by gambling, spending or having too much sex. Perhaps for this reason, ICDs are largely overlooked and poorly understood even today despite increasing attention and research over the last two decades. Nevertheless, anyone who has worked with patients suffering from one or more ICD or who is a sufferer himself will know exactly just how much guilt, shame, and functional disability there is. To support those in need, a comprehensive source about the frequency, evolution, treatment, related public policy, public health, forensic, and medical issues of these disorders is essential. However, to day, there have been limited resources available on both medical *and* social aspects of ICDs. *Impulse control disorders* attempts to fill this niche which makes this guide unique of its kind.

What do we know about impulse control disorders? What is their impact on society? How do cultural values facilitate and maintain the behaviour? Or, somewhat more practical questions: What are the financial costs of violence to society? How should kleptomania be addressed in court? This book aims to answer these, and many other exciting questions related to the most common impulse control disorders.

Perhaps the most obviously modern culture-related ICD of all is compulsive buying. As President Bush concluded after 9/11: “Mrs Bush and I want to encourage Americans to go out shopping”. Nowadays growth is about *having* more, not *being* more. It is therefore not surprising that the extent of “shopocalypse” urges attention.

The “having more” attitude may well be one of the reasons why theft is a major problem in the United States. The impulsive form of the behaviour, kleptomania, is estimated to account for about 5% of Americans charged with shoplifting annually. In general criminal responsibility is reduced because of the mental illness, however, kleptomans do not stand the cognitive test for insanity as they are very well aware of the illegal nature of their act. The authors report a curious court case from 1997 in Tennessee where a 47-year-old twice-divorced woman was accused of stealing $24.41 worth of merchandise from a store. She was sentenced to 11 months and 29 days in prison. The court argued that her lengthy history of shoplifting was a good-enough reason to protect the public from her further criminal acts...

Did you know that today Americans spend more on gambling than on any other form of entertainment? In the end, society pays the quantifiable costs which are estimated to be around $5 billion annually with an additional $40 billion productivity loss (which equals to Norway's export value in the same year, 1999!). Although gambling has officially become a public health issue, the activity is (un?)intentionally encouraged by many stakeholders across the world. Riverboat cruises, credit card organisations, media and the film industry, professional sports, even fund-raising bingo in schools send the message that it is okay to gamble.

Section II of the book describes the pellicular impulses. From the dermatologist's view there are three types of hair pullers: child, adult with insight and adult without insight hair pullers, all requiring different treatments. Affected hair-bearing areas often have a bizarre pattern with irregular borders and show a decreased density of hairs that are short but of varying length. A similar disorder, “psychodermatoses” or skin picking affects up to 2% of patients in dermatology clinics.

Another “modern addiction”, Internet addiction is listed under Section III: Information-seeking impulses. Does playing violent video games lead to violent behaviour? The authors argue that not having an answer to this question may indicate that the question has not been posed correctly in the first place.

Would you ever conduct counselling in the cyberspace? Would you ever accept an e-therapist? Thanks to modern technology, computer programs can be “trained” by recognised experts across a host of situations and nuances, modelling expert responses. Beyond the “perfect” answers, is there any chance we can ever train a computer to display empathy?

The last one, Section IV is about sexual and – perhaps the most problematic of all – aggressive impulses. Hyper-sexuality is presented from an unusual perspective: the sex industry's hidden victims' point of view. Because of their clients' sexual desires, sex workers are at especially high risk of violence, heroine use, STDS (higher risk than their clients!) and mental health issues. In addition, street sex work is criminalised in almost every part of the world (further victimisation by the authorities) which means added pressure to their already high-risk jobs. Sex work is a dangerous business.

Almost as dangerous as men suffering from intermittent explosive disorder and their violence against women. The United States has been classified as a rape-prone culture that celebrates aggression and eroticizes domination. Over 4.4 million women are physically assaulted each year by their intimate partners, 41% of which result in observable injuries. The National Institute of Justice calculated that costs *per victimization episode* for rape alone totalled more than $3 million for the year 1990, and the total annual cost of rape has been estimated a staggering $127 billion. This amount equals to the total US export to developing countries in the same year.

Another issue in the United States is intentional fire-setting. According to the statistics, there is a fire somewhere in every 20 seconds. Twenty percent of these fires are intentional fires and arson. In 2005, this resulted in 490 civilian fire deaths and 3 on-duty firefighter fatalities. Furthermore, there is publication to report that intentional fires and arson are likely to increase in bad economic times such as those being experienced in the United States (and around the world) currently. The other issue around arson is that half of all those arrested in the US are juveniles under the age of 18. Arson arrests account for a much larger proportion of arrests for youths under the age of 10 than any other crime that the FBI tracks (3%). Unfortunately, adults who present with fire-setting behaviour are usually directed to the criminal justice system with minimal or no contact with the mental health systems.

With impulse control disorders, overdose and fatalities are somewhat rare. However, their unfavourable effect on the individual's and society's level is more evident than ever. Elias Aboujaoude and Lorrin M. Koran edited a book which is not only informative and interesting to read, it is also eye-opening. They both have extensive experience with these disorders: Dr. Aboujaoude is the Director of the Impulse Disorders Clinic at Stanford University and Prof. Koran is the Director of the Obsessive–Compulsive Disorder Clinic at the same university. Chapters of the book are written by outstanding experts in the field of research and practice to provide first-hand information.

As a result, *Impulse control disorders* is not only a valuable source of information but also a well-written, interesting guide for researchers, clinicians or anyone who is interested in the personal and societal impact of these disorders beyond the mere symptoms.

