# Supplementary material for: Book Reviews
Source: J Behav Addict. 2013 Aug 20;2(3):187–9. doi: 10.1556/JBA.2.2013.3.9 (PMC4117290; doi:10.1556/JBA.2.2013.3.9)
Supplement: Related texts

# Sub article

**Authors:** Árpád Mihalik

**Affiliations:** Doctoral School of Psychology Eötvös Loránd University Budapest, Hungary



Nancy E. Willard, author of the book *Cyber-safe kids, cyber-savvy teens. Helping young people learn to use the Internet safely and responsibly,* is an expert in the field of cyberbullying and problematic Internet use among children and adolescents. Willard has a Bachelors of Science in Elementary and Early Childhood Education (University of Utah, 1975), a Masters of Science in Special Education (University of Oregon, 1977), and is a Doctor of Jurisprudence (Willamette University College of Law, 1983). As the director of the Center for Safe and Responsible Internet Use (CSRIU), Willard is aware of the issues of online risks concerning children and teenagers. The book *Cyber-safe kids, cyber-savvy teens* is dedicated to parents and teachers and supplies offline parenting and teaching methods.

On 324 pages the book discusses many topics such as privacy protection, the decision-making abilities of children and adolescents, online sexual behavior, cyberbullying, self-harm communities, online gaming and plagiarism. In each chapter there are examples of possible harm, some empirical results, technology-based advice and communication techniques. As a possible solution, Willard recommends the use of explicit rules. She suggests creating an agreement between parents and their children, one similar to the terms of agreements used by online sites. The appendix contains a template for “Parent–teen Internet use of agreement.” The agreement has to be made collectively and has to be accepted both by parents and their children. This contract – signed by both sides – must contain family values and standards regarding Internet use. Willard notes that important family values have to be reflected in Internet usage as well, in the same way as in any other offline habits. The book provides additional information that helps parents cooperate with their children to establish healthy Internet use, instead of making general restrictions.

As the title of the book suggests, Internet safety issues have to be discussed in a way which depends on the age of the user. Willard created five distinct groups differing in age: under 8, 8–10, 11–12, 13–15, 16–18, acknowledging that risks and concerns increase with the age of the user. This division is beneficial and practical, but perhaps it would have been even more fruitful if the reader received some more insight in the psychosocial features of the age clusters. Different methods, parental styles, and level of control are needed within the different age clusters to help the children and teens make safe choices in an online environment. It is only possible based on a trustworthy parental relationship to teach children and teens how to play safely and responsibly. The mutual reliance between parents and children creates ground for the gradual progress in which children become more autonomous, responsible and are in need of less adult control and monitoring.

The content of the book is divided into five chapters: 1) Internet parenting strategies, 2) Fundamental issues, 3) Influences on online decision making, 4) Specific risks and concerns, and 5) Accenting the positive.

The first chapter, *Internet parenting strategies* introduces the risks and concerns of Internet usage. Some of these are technical (hacking, copyright infringement, security concerns, etc.), while others are social problems (hate groups, sexual harassment, unsafe online communities, etc.) and the problem of addiction is also discussed (gambling, gaming, etc.). There is a short section about how parenting styles (authoritarian, permissive, authoritative) foster online and offline behavior. Perhaps it would have been a little more gainful if there was a wider empirical background about the mechanisms of different parenting styles and their effects in the family. This chapter introduces the interaction between age differences and methods, and the potential of a parent–teen contract which is a recurrent topic in many other chapters. One of the main goals of this chapter is to engage the reader to take responsibility for children and teens in the online environment. Willard shed light on the problems of technology based defense systems: softwares which over- or underblock certain webpages and weakness of the monitoring softwares. She concludes, however, that none of the protection softwares could replace a parent who takes responsibility for his or her child. Parents have to cooperate with children and teens by building knowledge, skills, and values which allow them to make consistently good choices on their own. Parents also have to cooperate with other parents and teachers to observe the rules laid down in the agreement at every place their children are able to use the Internet. The more adults are involved in the supervision of Internet usage, the less harm there may be. Last but not least, Willard sums up the potential warning signs of unsafe usage such as disturbed relationships, emotional upset and bullying.

Chapter 2 is about *Fundamental issues*. Willard provides further insight in the risks and dangers of specific Internet-related topics, such as commercials, privacy, Internet addiction, multitasking, information literacy, stranger literacy and the problematic features of social networking. Most of these issues are generated by the attributes of the online context. Teachers and parents have to be familiar with this new environment to prevent harmful impacts, and help improve children's skills to be able to make wise choices on their own. Willard guides the reader through the topic of social networking, e-commercials, privacy protection, Internet addiction, information and online stranger literacy as fundamental issues. Willard's most notable concern with social networking sites is the overrated social status of online relationships. The limitless and confusing online commercials and the undeveloped privacy protection strategies are also fundamental issues. Willard discusses Internet addiction and multi-tasking in one topic, after which she lists their indicators and suggests practical strategies to handle these problems. The last two fundamental issues in the chapter are information and online stranger literacy. These look familiar to traditional parenting patterns, but the online environment adds many new risk factors.

The next chapter discusses the background and the *Influences on online decision making*. Willard explains the basics of brain development which is responsible for decision making. By explaining that the age-based deficits (underdeveloped frontal lobe) of the brain regions is responsible for diminished decision making could help children understand that there are situations where they just have to ask for adult help. In addition to brain developmental difficulties, the online environment provides different context for making decisions. Willard mentions the problem of anonymity, the lower chance of detection and punishment, the lack of confrontation with actions in personal life, the possibility of social influence, and heavy social pressure from peers. These effects help rationalize decisions and have significant influence on online actions.

Chapter 4, called *Specific risks and concerns,* provides an in-depth discussion of each of the specific risks and concerns: 1) online pornography, 2) cyberbullying, 3) cyber-threats, 4) violent gaming, 5) gambling, 6) hate groups, 7) sites that support self-harm, 8) computer security concerns. Each of these topics starts with fictional stories about children and teens revealing how Internet-related problems effect their everyday life, or cause serious harm. After the illustrations, Willard defines the risks and concerns regarding the age difference of users, and the nature and frequency of the problems. She also gives parents recommendations for handling the emerging situations and talks about constructive ways of giving the knowledge, skills, and values to the children that can empower them to make the right choices online. Willard emphasizes the importance of a dialogue and the establishment of trust between parent and child. To handle the above-mentioned Internet-related problems, Willard gives practical advice on how to monitor, guide and effectively communicate with the child. It is worth noting, however, that most of these pieces of advice have not been empirically tested.

The last chapter of the book is about *Accenting the positive*. Even though the book highlights the negative aspects of the Internet, the last short chapter approaches the cyber-world as a potentially beneficial phenomenon. Willard notes that besides mindless surfing, endless gaming, or inane gambling, using the Internet can be very beneficial. The main advantages of Internet use according to Willard are: 1) fostering the communication within the family, 2) conducting research for school projects, 3) exploring subjects of personal interest, 4) creating own websites, 5) organizing teams and clubs, 6) discussing and proposing solutions to local issues, 6) organizing social servicing projects. Despite this summary of the positive possibilities, Willard has a quite negative judgment towards children's and teens' Internet use.

Willard's comprehensive book serves as a helping hand for parents and teachers who are in need of knowledge in the fields of online environments. Even though the methods and pieces of advice have not been empirically tested, they are likely to work. The main advantage of the book is that each chapter highlights the importance of making trustworthy relationship between parent and child. Establishing an honest relationship could be the most effective way to teach young people how to act safely and responsibly on their own, and make them feel responsible for themselves and others.
